# Supplementary material for: Effects of clozapine-N-oxide and compound 21 on sleep in laboratory mice
Source: eLife. 2023 Mar 9;12:e84740. doi: 10.7554/eLife.84740 (PMC9998087; doi:10.7554/eLife.84740)
Supplement: Supplementary file 1. [file elife-84740-supp1.docx]

**Supplementary Table 1: Time spent in wakefulness, NREM, and REM sleep after CNO and saline injections**

|  | | Treatment condition  (mean values ± standard error) | | | | Mixed-effect analysis | | Effect size for post-hoc comparisons (Cohen’s d) | | |
| --- | --- | --- | --- | --- | --- | --- | --- | --- | --- | --- |
| Vigilance state | Parameter | Saline (n=16) | CNO  1 mg/kg (n=11) | CNO  5 mg/kg (n=15) | CNO 10 mg/kg  (n=14) | *F* | *p* | low dose | medium dose | high dose |
| **Wake** |  |  |  |  |  |  |  |  |  |  |
|  | 2-h time window (%) | 38.7813  ±3.2743 | 38.3636  ±5.7692 | 33.5444  ±3.8399 | 29.6429  ±3.9910 | F (1.939, 23.91) = 1.468 | 0.2504 | -0.0234 | -0.3393 | -0.4815 |
|  | 6-h time window (%) | 28.3530  ±1.3114 | 29.3232  ±2.5037 | 26.9321  ±1.5278 | 26.2553  ±1.7923 | F (1.730, 21.34) = 0.6888 | 0.4928 | 0.1467 | -0.1715 | -0.2633 |
| **NREM** |  |  |  |  |  |  |  |  |  |  |
|  | 2-h time window (%) | 51.9896  ±2.6391 | 53.7020  ±4.6576 | 58.7741  ±3.2742 | 63.7103  ±3.7117 | F (1.911, 23.57) = 3.054 | 0.0682 | 0.1157 | 0.4941 | 0.6961 |
|  | 6-h time window (%) | 59.0799  ±1.1641 | 58.9074  ±1.9870 | 62.1914  ±1.2667 | 62.6111  ±1.6784 | F (1.531, 18.88) = 2.028 | 0.1661 | -0.0361 | 0.4130 | 0.4630 |
| **REM** |  |  |  |  |  |  |  |  |  |  |
|  | 2-h time window  (% of TST) | 10.4160  ±0.5877 | 8.1286  ±1.0534 | 8.4071  ±0.8351 | 6.5371  ±0.5937 | F (1.783, 21.99) = 8.951 | 0.0019 | -0.5802 | -0.5594 | -1.2743 |
|  | 6-h time window  (% of TST) | 13.1740  ±0.3774 | 12.3119  ±0.4884 | 11.1629  ±0.5722 | 11.0742  ±0.4606 | F (1.839, 22.68) = 7.525 | 0.0038 | -0.4697 | -0.7724 | -0.9333 |
|  | 2-h time window (% of NREM) | 11.6996  ±0.7374 | 8.9862  ±1.2086 | 9.3021  ±0.9672 | 7.0497  ±0.6714 | F (1.858, 22.91) = 9.140 | 0.0015 | -0.5895 | -0.5673 | -1.2707 |
|  | 6-h time window (% of NREM) | 15.2055  ±0.5001 | 14.0760  ±0.6364 | 12.6328  ±0.7455 | 12.4919  ±0.5741 | F (1.788, 22.05) = 7.382 | 0.0045 | -0.4658 | -0.7509 | -0.9430 |
|  | 2-h time window (%) | 6.1354  ±0.5709 | 5.2121  ±0.8723 | 5.5296  ±0.6426 | 4.3492  ±0.4418 | F (2.366, 29.18) = 2.590 | 0.0839 | -0.3251 | -0.2348 | -0.6559 |
|  | 6-h time window (%) | 8.9329  ±0.2401 | 8.2963  ±0.4838 | 7.8568  ±0.4849 | 7.7619  ±0.3314 | F (2.350, 28.99) = 2.959 | 0.0601 | -0.3718 | -0.5591 | -0.8140 |
