# Supplementary material for: Effects of clozapine-N-oxide and compound 21 on sleep in laboratory mice
Source: eLife. 2023 Mar 9;12:e84740. doi: 10.7554/eLife.84740 (PMC9998087; doi:10.7554/eLife.84740)
Supplement: Supplementary file 2. [file elife-84740-supp2.docx]

**Supplementary Table 2: Sleep architecture after CNO and saline injections**

|  | | Treatment condition  (mean values ± standard error) | | | | Mixed-effect analysis | | Effect size for post-hoc comparisons (Cohen’s d) | | |
| --- | --- | --- | --- | --- | --- | --- | --- | --- | --- | --- |
| Vigilance state | Parameter | Saline (n=16) | CNO  1 mg/kg (n=11) | CNO  5 mg/kg (n=15) | CNO 10 mg/kg  (n=14) | *F* | *p* | low dose | medium dose | high dose |
| **Wake** |  |  |  |  |  |  |  |  |  |  |
|  | Longest episode (min) | 31.3292±2.3901 | 32.0000±3.9528 | 31.6089±3.2855 | 31.7952 ±3.5150 | F (2.108, 26.00) = 0.02110 | 0.9825 | 0.0663 | 0.0189 | 0.0298 |
|  | Episode duration average (min) | 11.6562  ±0.9793 | 9.6981  ±0.6636 | 11.0855  ±0.8599 | 11.0939  ±0.9275 | F (2.578, 31.80) = 0.8177 | 0.4777 | -0.4160 | -0.1221 | -0.1355 |
|  | Episode number (n/h) | 1.5313 ±0.1429 | 1.7727 ±0.1107 | 1.4889 ±0.1254 | 1.4286 ±0.0984 | F (2.338, 40.52) = 1.241 | 0.3036 | 0.3046 | -0.0592 | -0.1694 |
| **NREM** |  |  |  |  |  |  |  |  |  |  |
|  | Longest episode (min) | 16.3458  ±0.6994 | 18.8061  ±1.6012 | 22.1067  ±1.1553 | 26.0905  ±1.5547 | F (2.881, 35.53) = 13.24 | <0.0001 | 0.3562 | 0.9438 | 1.5129 |
|  | Episode duration average (min) | 5.8746  ±0.2822 | 6.1381  ±0.2998 | 7.2894  ±0.3127 | 7.5503  ±0.4268 | F (2.436, 30.04) = 11.64 | <0.0001 | 0.1810 | 1.1430 | 1.1883 |
|  | Episode number (n/h) | 6.3021  ±0.2318 | 5.9697  ±0.3113 | 5.3556  ±0.2444 | 5.2857  ±0.2915 | F (2.476, 30.54) = 7.796 | 0.0010 | -0.2938 | -1.0628 | -1.1989 |
|  | Latency (min) | 30.4958±2.9506 | 14.6485±2.9015 | 26.1378±3.3676 | 25.4762 ±4.6781 | F (2.146, 26.47) = 3.380 | 0.0463 | -1.2021 | -0.3744 | -0.2424 |
| **REM** |  |  |  |  |  |  |  |  |  |  |
|  | Longest episode (min) | 3.2417  ±0.1481 | 3.2364  ±0.1699 | 3.3378  ±0.1291 | 3.4190  ±0.1546 | F (2.605, 32.12) = 0.5517 | 0.6266 | -0.0083 | 0.1710 | 0.2450 |
|  | Episode duration average (min) | 1.2260  ±0.0415 | 1.1620  ±0.0456 | 1.3546  ±0.0399 | 1.3442  ±0.0651 | F (2.150, 26.52) = 4.230 | 0.0232 | -0.3757 | 0.5463 | 0.4111 |
|  | Episode number (n/h) | 4.3958  ±0.1747 | 4.3182  ±0.2934 | 3.5333  ±0.2644 | 3.5357  ±0.2245 | F (2.124, 26.19) = 6.430 | 0.0047 | -0.0892 | -0.7456 | -0.8710 |
|  | Latency (min) | 22.2000  ±2.8840 | 46.0485  ±9.5971 | 28.9689  ±6.1401 | 34.8952  ±3.0666 | F (1.840, 22.69) = 4.691 | 0.0220 | 0.8061 | 0.3202 | 0.9213 |
| **Sleep consoli-dation** |  |  |  |  |  |  |  |  |  |  |
|  | NREM before REM onset (min) | 16.1333±1.307 | 25.3333±3.7126 | 23.5956±3.4938 | 28.4524 ±2.0227 | F (2.031, 25.04) = 5.087 | 0.0137 | 0.7996 | 0.5317 | 1.5467 |
|  | Brief awakenings (n/h)* | 36.8033±3.7206 | 27.6169±3.2139 | 20.9897±2.7844 | 21.5082±2.0680 | F (1.968, 23.62) = 10.38 | 0.0006 | -0.6271 | -1.0691 | -1.5760 |

*animal numbers for analysis of brief awakenings: n=15 for saline, n=11 for 1 mg/kg, n=15 for 5 mg/kg, n=13 for 10 mg/kg and time window 2h.
