# Supplementary material for: Effects of clozapine-N-oxide and compound 21 on sleep in laboratory mice
Source: eLife. 2023 Mar 9;12:e84740. doi: 10.7554/eLife.84740 (PMC9998087; doi:10.7554/eLife.84740)
Supplement: Supplementary file 3. [file elife-84740-supp3.docx]

**Supplementary Table 3: NREM sleep architecture for the first 2 hours after CNO and saline injections**

|  | | Treatment condition  (mean values ± standard error) | | | | Mixed-effect analysis | | Effect size for post-hoc comparisons (Cohen’s d) | | |
| --- | --- | --- | --- | --- | --- | --- | --- | --- | --- | --- |
| Vigilance state | Parameter | Saline (n=16) | CNO  1 mg/kg (n=11) | CNO  5 mg/kg (n=15) | CNO 10 mg/kg  (n=14) | *F* | *p* | low dose | medium dose | high dose |
| **NREM** |  |  |  |  |  |  |  |  |  |  |
|  | Longest episode (min) | 14.0875  ±0.8735 | 16.6424  ±1.7807 | 20.8089  ±1.0784 | 24.2524  ±1.8227 | F (2.144, 26.44) = 12.43 | 0.0001 | 0.3030 | 1.6520 | 1.4053 |
|  | Episode duration average (min) | 6.7120  ±0.4205 | 7.8408  ±0.9216 | 9.8579  ±0.5317 | 10.1328  ±0.4766 | F (2.388, 29.45) = 9.042 | 0.0005 | 0.2847 | 1.0681 | 1.1462 |
|  | Episode number (n/h) | 5.0938  ±0.4139 | 4.7727  ±0.6229 | 3.7667  ±0.2797 | 3.9643  ±0.3251 | F (2.031, 25.05) = 3.391 | 0.0491 | -0.1375 | -0.8787 | -0.5501 |
