# Supplementary material for: Effects of clozapine-N-oxide and compound 21 on sleep in laboratory mice
Source: eLife. 2023 Mar 9;12:e84740. doi: 10.7554/eLife.84740 (PMC9998087; doi:10.7554/eLife.84740)
Supplement: Supplementary file 4. [file elife-84740-supp4.docx]

**Supplementary Table 4: ANOVA results for EEG spectra following CNO and saline injections**

| Vigilance state | Conditions | Time window | Main effect | *F* | *p* |
| --- | --- | --- | --- | --- | --- |
| **Wake** |  |  |  |  |  |
|  | 1 mg/kg vs. saline | 2 hours | ‘condition’ | *F*_(1,5)_ = 0.06176 | 0.8136 |
|  |  |  | ‘frequency’ x ‘condition’ | *F*_(118,590)_ = 1.137 | 0.1720 |
|  |  | 6 hours | ‘condition’ | *F*_(1,5)_ = 0.03580 | 0.8574 |
|  |  |  | ‘frequency’ x ‘condition’ | *F*_(118,590)_ = 0.6646 | 0.9966 |
|  | 5 mg/kg vs. saline | 2 hours | ‘condition’ | *F*_(1,9)_ = 0.7492 | 0.4092 |
|  |  |  | ‘frequency’ x ‘condition’ | *F*_(118,1062)_ = 0.5995 | 0.9997 |
|  |  | 6 hours | ‘condition’ | *F*_(1,9)_ = 0.0006565 | 0.9801 |
|  |  |  | ‘frequency’ x ‘condition’ | *F*_(118,1062)_ = 0.9777 | 0.5507 |
|  | 10 mg/kg vs. saline | 2 hours | ‘condition’ | *F*_(1,7)_ = 4.602 | 0.0691 |
|  |  |  | ‘frequency’ x ‘condition’ | *F*_(118, 826)_ = 0.8017 | 0.9344 |
|  |  | 6 hours | ‘condition’ | *F*_(1,7)_ = 3.232 | 0.1153 |
|  |  |  | ‘frequency’ x ‘condition’ | *F*_(118, 826)_ = 1.130 | 0.1778 |
| **NREM** |  |  |  |  |  |
|  | 1 mg/kg vs. saline | 2 hours | ‘condition’ | *F*_(1,5)_ = 0.1893 | 0.6816 |
|  |  |  | ‘frequency’ x ‘condition’ | *F*_(118,590)_ = 0.8900 | 0.7805 |
|  |  | 6 hours | ‘condition’ | *F*_(1,5)_ = 0.1227 | 0.7404 |
|  |  |  | ‘frequency’ x ‘condition’ | *F*_(118,590)_ = 0.9481 | 0.6329 |
|  | 5 mg/kg vs. saline | 2 hours | ‘condition’ | *F*_(1,9)_ = 17.80 | 0.0022 |
|  |  |  | ‘frequency’ x ‘condition’ | *F*_(118,1062)_ = 10.83 | <0.0001 |
|  |  | 6 hours | ‘condition’ | *F*_(1,9)_ = 10.99 | 0.0090 |
|  |  |  | ‘frequency’ x ‘condition’ | *F*_(118, 1062)_ = 4.726 | <0.0001 |
|  | 10 mg/kg vs. saline | 2 hours | ‘condition’ | *F*_(1,7)_ = 13.01 | 0.0087 |
|  |  |  | ‘frequency’ x ‘condition’ | *F*_(118, 826)_ = 5.798 | <0.0001 |
|  |  | 6 hours | ‘condition’ | *F*_(1,7)_ = 12.30 | 0.0099 |
|  |  |  | ‘frequency’ x ‘condition’ | *F*_(118, 826)_ = 7.274 | <0.0001 |
| **REM** |  |  |  |  |  |
|  | 1 mg/kg vs. saline | 2 hours | ‘condition’ | *F*_(1,4)_ = 1.232 | 0.3292 |
|  |  |  | ‘frequency’ x ‘condition’ | *F*_(118,472)_ = 0.9809 | 0.5410 |
|  |  | 6 hours | ‘condition’ | *F*_(1,5)_ = 6.219 | 0.0549 |
|  |  |  | ‘frequency’ x ‘condition’ | *F*_(118,590)_ = 1.372 | 0.0100 |
|  | 5 mg/kg vs. saline | 2 hours | ‘condition’ | *F*_(1,8)_ = 3.119 | 0.1154 |
|  |  |  | ‘frequency’ x ‘condition’ | *F*_(118,944)_ = 0.7339 | 0.9828 |
|  |  | 6 hours | ‘condition’ | *F*_(1,9)_ = 1.966 | 0.1944 |
|  |  |  | ‘frequency’ x ‘condition’ | *F*_(118, 1062)_ = 1.229 | 0.0574 |
|  | 10 mg/kg vs. saline | 2 hours | ‘condition’ | *F*_(1,7)_ = 0.007106 | 0.9352 |
|  |  |  | ‘frequency’ x ‘condition’ | *F*_(118, 826)_ = 1.298 | 0.0243 |
|  |  | 6 hours | ‘condition’ | *F*_(1,7)_ = 0.0002323 | 0.9883 |
|  |  |  | ‘frequency’ x ‘condition’ | *F*_(118, 826)_ = 2.327 | <0.0001 |
