# Supplementary material for: Effects of clozapine-N-oxide and compound 21 on sleep in laboratory mice
Source: eLife. 2023 Mar 9;12:e84740. doi: 10.7554/eLife.84740 (PMC9998087; doi:10.7554/eLife.84740)
Supplement: Supplementary file 5. [file elife-84740-supp5.docx]

**Supplementary Table 5: Sleep parameters after saline and compound 21 injections**

| Vigilance state | Parameter | Saline (n=7) | C21 3 mg/kg  (n=7) | *t* | *p* | Effect size (Cohen’s d) |
| --- | --- | --- | --- | --- | --- | --- |
| **Wake** |  |  |  |  |  |  |
|  | 2-h time window (%) | 31.4841  ±4.1581 | 29.3571  ±3.7073 | t=0.2993, df=6 | 0.3874 | -0.1131 |
|  | 6-h time window (%) | 26.4153  ±2.1399 | 23.5106  ±1.9486 | t=0.9230, df=6 | 0.1958 | -0.3489 |
|  | Longest episode (min) | 27.6286  ±2.5082 | 30.3524  ±4.1173 | t=0.8184, df=6 | 0.2222 | 0.3093 |
|  | Episode duration average (min) | 10.4205  ±0.9875 | 10.6269  ±1.8085 | t=0.1339, df=6 | 0.4489 | 0.0506 |
|  | Episode number (n/h) | 1.5714  ±0.2463 | 1.4286  ±0.1776 | t=0.4680, df=6 | 0.3281 | -0.1769 |
| **NREM** |  |  |  |  |  |  |
|  | 2-h time window (%) | 58.9444  ±2.9443 | 63.2302  ±3.0788 | t=0.8081, df=6 | 0.2249 | 0.3054 |
|  | 6-h time window (%) | 61.4603  ± 1.8489 | 65.5873  ±1.6922 | t=1.623, df=6 | 0.0779 | 0.6133 |
|  | Latency (min) | 25.3619  ±3.2217 | 22.6571  ±4.8946 | t=0.3844, df=6 | 0.3570 | -0.1453 |
|  | Longest episode (min) | 15.9143  ±0.7453 | 21.5429  ±1.6727 | t=2.551, df=6 | 0.0217 | 0.9641 |
|  | Episode duration average (min) | 6.0655  ±0.5040 | 8.0568  ±0.5341 | t=2.462, df=6 | 0.0245 | 0.9307 |
|  | Episode number (n/h) | 6.3571  ±0.2708 | 5.1190  ±0.3426 | t=2.809, df=6 | 0.0154 | -1.0617 |
| **REM** |  |  |  |  |  |  |
|  | 2-h time window  (% of TST) | 9.9182  ± 0.9511 | 7.5927  ±0.3919 | t=1.829, df=6 | 0.0585 | -0.6915 |
|  | 6-h time window  (% of TST) | 12.6278  ±0.5749 | 10.8374  ±0.3969 | t=3.234, df=6 | 0.0089 | -1.2223 |
|  | 2-h time window  (% of NREM) | 11.0846  ±1.1739 | 8.2282  ±0.4615 | t=1.845, df=6 | 0.0573 | -0.6972 |
|  | 6-h time window  (% of NREM) | 14.4824  ±0.7491 | 12.1680  ±0.5030 | t=3.253, df=6 | 0.0087 | -1.2296 |
|  | 2-h time window (%) | 6.6508  ±0.9090 | 5.2460  ±0.4823 | t=1.050, df=6 | 0.1670 | -0.3970 |
|  | 6-h time window (%) | 8.8571  ±0.3862 | 7.9630  ±0.3277 | t=2.086, df=6 | 0.0410 | -0.7885 |
|  | Latency (min) | 18.2667±3.1860 | 28.1524  ±3.2043 | t=2.043, df=6 | 0.0435 | 0.7722 |
|  | Longest episode (min) | 3.3048  ±0.1664 | 3.4667  ±0.2718 | t=0.5265, df=6 | 0.3087 | 0.1990 |
|  | Episode duration average (min) | 1.2433  ±0.0706 | 1.5442  ±0.1048 | t=2.082, df=6 | 0.0413 | 0.7869 |
|  | Episode number (n/h) | 4.2857  ±0.2432 | 3.1429  ±0.1939 | t=4.942, df=6 | 0.0013 | -1.8679 |
| **Sleep consoli-dation** |  |  |  |  |  |  |
|  | NREM before REM onset (min) | 15.5238  ±2.2464 | 22.8190  ±1.9541 | t=4.092, df=6 | 0.0032 | 1.5467 |
|  | Brief awakenings (n/h)* | 31.5102  ±2.6435 | 20.1852  ±2.6435 | t=2.164, df=5 | 0.0414 | -0.8836 |

*animal numbers for analysis of brief awakenings: n=6 and time window: 2 hours.
