# Supplementary material for: Effects of clozapine-N-oxide and compound 21 on sleep in laboratory mice
Source: eLife. 2023 Mar 9;12:e84740. doi: 10.7554/eLife.84740 (PMC9998087; doi:10.7554/eLife.84740)
Supplement: Supplementary file 6. [file elife-84740-supp6.docx]

**Supplementary Table 6: NREM sleep architecture for the first 2 hours after C21 and saline injections**

| Vigilance state | Parameter | Saline (n=7) | C21 3 mg/kg  (n=7) | *t* | *p* | Effect size (Cohen’s d) |
| --- | --- | --- | --- | --- | --- | --- |
| **NREM** |  |  |  |  |  |  |
|  | Longest episode (min) | 14.5238  ±1.0721 | 19.6381  ±1.2726 | t=2.601, df=6 | 0.0203 | 0.9831 |
|  | Episode duration average (min) | 6.8074  ±0.6933 | 11.5585  ±1.4770 | t=2.222, df=6 | 0.0340 | 0.8398 |
|  | Episode number (n/h) | 5.6429  ±0.5639 | 3.7857  ±0.5654 | t=1.705, df=6 | 0.0696 | -0.6443 |
