# Supplementary material for: Effects of clozapine-N-oxide and compound 21 on sleep in laboratory mice
Source: eLife. 2023 Mar 9;12:e84740. doi: 10.7554/eLife.84740 (PMC9998087; doi:10.7554/eLife.84740)
Supplement: MDAR checklist [file elife-84740-mdarchecklist1.docx]

**Materials Design Analysis Reporting (MDAR)**

**Checklist for Authors**

The [MDAR framework](https://osf.io/xfpn4/) establishes a minimum set of requirements in transparent reporting mainly applicable to studies in the life sciences.

*eLife* asks authors to **provide detailed information within their article** to facilitate the interpretation and replication of their work. Authors can also upload supporting materials to comply with relevant reporting guidelines for health-related research (see [EQUATOR Network](http://www.equator-network.org/%20)), life science research (see the [BioSharing Information Resource](http://biosharing.org/)), or animal research (see the [ARRIVE Guidelines](http://www.plosbiology.org/article/info:doi/10.1371/journal.pbio.1000412) and the [STRANGE Framework](https://doi.org/10.1038/d41586-020-01751-5); for details, see *eLife*’s [Journal Policies](https://reviewer.elifesciences.org/author-guide/journal-policies)). Where applicable, authors should refer to any relevant reporting standards materials in this form.

For all that apply, please note **where in the article** the information is provided. Please note that we also collect information about data availability and ethics in the submission form.

**Materials:**

| **Newly created materials** | **Indicate where provided: section/figure legend** | **N/A** |
| --- | --- | --- |
| The manuscript includes a dedicated "materials availability statement" providing transparent disclosure about availability of newly created materials including details on how materials can be accessed and describing any restrictions on access. | Please see **manuscript section “Data and code availability”**:  “Pre-processed raw data in the form of Matlab files with frontal EEG spectra and vigilance state annotation for each 4s-epoch of all individual days and all animals as well as custom written MATLAB code, and GraphPad Prism data tables for all figures are available on Figshare (private link provided in point-to-point response to the reviewers. Repository will be made openly available upon acceptance of the manuscript). EEG/EMG data tanks for all recordings are too large for conventional online repositories, but are freely available upon reasonable request.”  For an example of a Figshare project from our research group, please refer to our recent publication Krone et al., *Nature Neuroscience* (2021). |  |
|  |  |  |
| **Antibodies** | **Indicate where provided: section/figure legend** | **N/A** |
| For commercial reagents, provide supplier name, catalogue number and [RRID](https://scicrunch.org/resources), if available. |  | x |
|  |  |  |
| **DNA and RNA sequences** | **Indicate where provided: section/figure legend** | **N/A** |
| Short novel DNA or RNA including primers, probes: Sequences should be included or deposited in a public repository. |  | x |
|  |  |  |
| **Cell materials** | **Indicate where provided: section/figure legend** | **N/A** |
| Cell lines: Provide species information, strain. Provide accession number in repository OR supplier name, catalog number, clone number, OR RRID. |  | x |
| Primary cultures: Provide species, strain, sex of origin, genetic modification status. |  | x |
|  |  |  |
| **Experimental animals** | **Indicate where provided: section/figure legend** | **N/A** |
| Laboratory animals or Model organisms: Provide species, strain, sex, age, genetic modification status. Provide accession number in repository OR supplier name, catalog number, clone number, OR RRID. | Please see **manuscript section “Animals”**. |  |
| Animal observed in or captured from the field: Provide species, sex, and age where possible. |  | x |
|  |  |  |
| **Plants and microbes** | **Indicate where provided: section/figure legend** | **N/A** |
| Plants: provide species and strain, ecotype and cultivar where relevant, unique accession number if available, and source (including location for collected wild specimens). |  | x |
| Microbes: provide species and strain, unique accession number if available, and source. |  | x |
|  |  |  |
| **Human research participants** | **Indicate where provided: section/figure legend) or state if these demographics were not collected** | **N/A** |
| If collected and within the bounds of privacy constraints report on age, sex, gender and ethnicity for all study participants. |  | **x** |

**Design:**

| **Study protocol** | **Indicate where provided: section/figure legend** | **N/A** |
| --- | --- | --- |
| If the study protocol has been pre-registered, provide DOI. For clinical trials, provide the trial registration number OR cite DOI. |  | x |
|  |  |  |
| **Laboratory protocol** | **Indicate where provided: section/figure legend** | **N/A** |
| Provide DOI OR other citation details if detailed step-by-step protocols are available. |  | x |
|  |  |  |
| **Experimental study design (statistics details) *** | | |
| **For in vivo studies: State whether and how the following have been done** | **Indicate where provided: section/figure legend. If it could have been done, but was not, write “not done”** | **N/A** |
| Sample size determination | YES, sample size determination was performed using previous data from our lab on pharmacological manipulation of sleep (McKillop et al., *Biochemical Pharmacology* (2021)).  For more details, please see **manuscript section: “Sample size determination and power analysis”** |  |
| Randomisation | YES, animals were randomly allocated to a **semi-counterbalanced study design for the CNO conditions**.  Please note that there was **no randomization or counterbalancing for the C21** condition. For more details, please see **manuscript section:  “Experimental design”** |  |
| Blinding | YES, **experimenters were blinded to the experimental condition during sleep scoring**. Blinding of the experimenters during the injection process was not possible as the solutions of the chemical actuators have a light yellow colour and hence can be distinguished from pure saline with the naked eye.  For more details, please see **manuscript section: “Electrophysiological signal acquisition, data processing, and sleep scoring”** |  |
| Inclusion/exclusion criteria | **No animal was excluded from data analysis**. **Exclusion of a subset of recorded data was necessary for individual analyses due to technical issues.** E.g. EEG recordings with electrical artefacts could be used for vigilance state and sleep architecture analysis but had to be excluded from spectral analysis. Detailed information is provided in **manuscript section: “Statistical procedures”**. |  |
|  |  |  |
| **Sample definition and in-laboratory replication** | **Indicate where provided: section/figure legend** | **N/A** |
| State number of times the experiment was replicated in the laboratory. | The **sixteen animals** used for this study were **recorded in four batches.** |  |
| Define whether data describe technical or biological replicates. | **Both biological and technical replicates were used**. Different animals represent biological replicates, different injections (e.g. different CNO doses in the same animal) represent technical replicates. For more details, please see **manuscript section “Experimental design”**. |  |
|  |  |  |
| **Ethics** | **Indicate where provided: section/submission form** | **N/A** |
| Studies involving human participants: State details of authority granting ethics approval (IRB or equivalent committee(s), provide reference number for approval. |  | x |
| Studies involving experimental animals: State details of authority granting ethics approval (IRB or equivalent committee(s), provide reference number for approval. | For details on the ethical approval please see **manuscript section: “Ethical approvals”**. The project was conducted on the **United Kingdom Home Office project license number P828B64BC**. |  |
| Studies involving specimen and field samples: State if relevant permits obtained, provide details of authority approving study; if none were required, explain why. |  | x |
|  |  |  |
| **Dual Use Research of Concern (DURC)** | **Indicate where provided: section/submission form** | **N/A** |
| If study is subject to dual use research of concern regulations, state the authority granting approval and reference number for the regulatory approval. |  | x |

**Analysis:**

| **Attrition** | **Indicate where provided: section/figure legend** | **N/A** |
| --- | --- | --- |
| Describe whether exclusion criteria were pre-established. Report if sample or data points were omitted from analysis. If yes, report if this was due to attrition or intentional exclusion and provide justification. | YES, exclusion criteria were identical to several previous studies from our lab and other research groups performing sleep research in rodents using *in vivo* electrophysiology. **No data points were omitted at the analysis stage**. **Few individual data files were considered unsuitable for specific analyses due to technical issues** encountered during the experiment (e.g. documented backflow of injected drug immediately after injection in one condition in one animal) or discovered during preprocessing (i.e. electrical artefacts identified during data import and sleep scoring). A detailed justification is provided in **manuscript section “Statistical procedures”**. |  |
|  |  |  |
| **Statistics** | **Indicate where provided: section/figure legend** | **N/A** |
| Describe statistical tests used and justify choice of tests. | For statistical comparison of clozapine-N-oxide (CNO) and saline injections we used mixed-effect models. This test appeared most suitable to assess the effect of repeated injections in the same animals while not all animals could receive all injections. For the statistical comparison of compound 21 (C21) and saline injections we used one-sided t-tests for paired samples. The rational for this test was that we were aware of the low statistical power in a group of n=7 animals and that we had a clear expectation about the directionality of the effect (i.e. the same direction in the CNO condition). As the usefulness of applying null-hypothesis testing in general, and one-sided t-tests in particular, to this small dataset is certainly controversial, we also provide effect sizes (Cohen’s d) and confidence intervals for each analysis as well as source data tables to enable re-analysis of and application of a different statistical test to this data set. For more details as well as information about analysis of electroencephalogram (EEG) spectral data, please see **section “Statistical procedures”**. |  |
|  |  |  |
| **Data availability** | **Indicate where provided: section/submission form** | **N/A** |
| For newly created and reused datasets, the manuscript includes a data availability statement that provides details for access (or notes restrictions on access). | **All data will be made publicly available on a Figshare repository**. For more information, please see manuscript section: “Data and code availability”. |  |
| When newly created datasets are publicly available, provide accession number in repository OR DOI and licensing details where available. | A DOI for the Figshare project with a CC BY 4.0 international public license has been reserved. **The DOI will be made publicly available upon acceptance of the manuscript.**  For an example of a Figshare project from our research group, please refer to our recent publication Krone et al., *Nature Neuroscience* (2021). |  |
| If reused data is publicly available provide accession number in repository OR DOI, OR URL, OR citation. | **The data used in this project was newly collected.** No reused data was included. |  |
|  |  |  |
| **Code availability** | **Indicate where provided: section/figure legend** | **N/A** |
| For any computer code/software/mathematical algorithms essential for replicating the main findings of the study, whether newly generated or re-used, the manuscript includes a data availability statement that provides details for access or notes restrictions. | Matlab code for data processing as well as GraphPad Prism files for statistical analysis **will be made publicly available on Figshare** with a CC BY 4.0 international public license and DOI upon acceptance of the manuscript. |  |
| Where newly generated code is publicly available, provide accession number in repository, OR DOI OR URL and licensing details where available. State any restrictions on code availability or accessibility. | **A DOI for the Figshare project with a CC BY 4.0 international public licenses will be provided upon acceptance of the manuscript.**  For an example of a Figshare repository from our research group, please refer to our recent publication Krone et al., *Nature Neuroscience* (2021). |  |
| If reused code is publicly available provide accession number in repository OR DOI OR URL, OR citation. | **No publicly available code was reused** in this study. |  |

**Reporting:**

The MDAR framework recommends adoption of discipline-specific guidelines, established and endorsed through community initiatives.

| **Adherence to community standards** | **Indicate where provided: section/figure legend** | **N/A** |
| --- | --- | --- |
| State if relevant guidelines (e.g., ICMJE, MIBBI, ARRIVE, STRANGE) have been followed, and whether a checklist (e.g., CONSORT, PRISMA, ARRIVE) is provided with the manuscript. | **The ARRIVE guidelines (Percie du Sert et al., *PLOS Biology* (2020)) have been followed throughout the course of this study and in the preparation of this manuscript**. We do not provide a checklist with the manuscript to avoid redundancy of information with the details provided in the methods section and in this MDAR checklist. |  |

* We provide the following guidance regarding transparent reporting and statistics; we also refer authors to [Ten common statistical mistakes to watch out for when writing or reviewing a manuscript](https://doi.org/10.7554/eLife.48175).

**Sample-size estimation**

- You should state whether an appropriate sample size was computed when the study was being designed
- You should state the statistical method of sample size computation and any required assumptions
- If no explicit power analysis was used, you should describe how you decided what sample (replicate) size (number) to use

**Replicates**

- You should report how often each experiment was performed
- You should include a definition of biological versus technical replication
- The data obtained should be provided and sufficient information should be provided to indicate the number of independent biological and/or technical replicates
- If you encountered any outliers, you should describe how these were handled
- Criteria for exclusion/inclusion of data should be clearly stated
- High-throughput sequence data should be uploaded before submission, with a private link for reviewers provided (these are available from both GEO and ArrayExpress)

**Statistical reporting**

- Statistical analysis methods should be described and justified
- Raw data should be presented in figures whenever informative to do so (typically when N per group is less than 10)
- For each experiment, you should identify the statistical tests used, exact values of N, definitions of center, methods of multiple test correction, and dispersion and precision measures (e.g., mean, median, SD, SEM, confidence intervals; and, for the major substantive results, a measure of effect size (e.g., Pearson's r, Cohen's d)
- Report exact p-values wherever possible alongside the summary statistics and 95% confidence intervals. These should be reported for all key questions and not only when the p-value is less than 0.05.

**Group allocation**

- Indicate how samples were allocated into experimental groups (in the case of clinical studies, please specify allocation to treatment method); if randomization was used, please also state if restricted randomization was applied
- Indicate if masking was used during group allocation, data collection and/or data analysis
